# Redox-Active Selenium Compounds—From Toxicity and Cell Death to Cancer Treatment

**DOI:** 10.3390/nu7053536

**Published:** 2015-05-13

**Authors:** Sougat Misra, Mallory Boylan, Arun Selvam, Julian E. Spallholz, Mikael Björnstedt

**Affiliations:** 1 Division of Pathology F46, Department of Laboratory Medicine, Karolinska Institutet, Karolinska University Hospital Huddinge, Stockholm 141 86, Sweden; E-Mails: sougat.misra@ki.se (S.M.); arun.selvam@ki.se (A.S.); 2 Department of Nutritional Sciences, College of Human Sciences, Texas Tech University, P.O. Box 41270, Lubbock, TX 79409-1270, USA; E-Mails: mallory.boylan@ttu.edu (M.B.); Julian.Spallholz@ttu.edu (J.E.S.)

**Keywords:** selenium, antioxidants, selenoproteins, cancer, tumor, cytotoxicity

## Abstract

Selenium is generally known as an antioxidant due to its presence in selenoproteins as selenocysteine, but it is also toxic. The toxic effects of selenium are, however, strictly concentration and chemical species dependent. One class of selenium compounds is a potent inhibitor of cell growth with remarkable tumor specificity. These redox active compounds are pro-oxidative and highly cytotoxic to tumor cells and are promising candidates to be used in chemotherapy against cancer. Herein we elaborate upon the major forms of dietary selenium compounds, their metabolic pathways, and their antioxidant and pro-oxidant potentials with emphasis on cytotoxic mechanisms. Relative cytotoxicity of inorganic selenite and organic selenocystine compounds to different cancer cells are presented as evidence to our perspective. Furthermore, new novel classes of selenium compounds specifically designed to target tumor cells are presented and the potential of selenium in modern oncology is extensively discussed.

## 1. Introduction

The common feature among selenium compounds inhibiting the growth of neoplastic cells is their pronounced and unique redox activity either by the compound itself or by metabolites, where some selenium compounds, as exemplified by Se-methylselenocysteine, are to be considered as prodrugs. This redox property is essential for selenium’s antineoplastic effects and the application of selenium compounds in cancer therapeutics. Naturally occurring selenium compounds in possession of these properties are selenite, selenocysteine, other selenolates (RSe¯) and the methylselenol metabolite (CH_3_Se¯) of Se-methylselenocysteine and selenomethionine. There is also a new generation of compounds available and under development that will efficiently deliver redox active cytotoxic selenium to tumor cells. These new compounds, together with the well-known natural dietary supplemental compounds, will be discussed in this review. The cytotoxic effects of all selenium compounds with effects against tumor cells are dependent on the appearance and regeneration of free selenols and selenolates, either chemically by the oxidation of any accessible thiols, protein bound or free (e.g., glutathione), or enzymatically generated by reductases as exemplified by the thioredoxin and glutaredoxin systems [1,2,3]. The selenolates and hydrogen selenide (HSe¯) efficiently react with oxygen and thiols leading to a non-stoichiometric consumption of thiols and NADPH, massive oxidative stress and eventually cell death due to apoptosis, necrosis or necroptosis [2,3,4].

The usual physiological and nutritional properties and functions of selenium are commonly recognized. There is, though, a major difference between selenium compounds and other trace elements, vitamins and antioxidants, since selenium compounds can be extremely toxic, a property that will be further elaborated upon in this review. This inherent, concentration and redox dependent, toxicity is the basis for the unique anti-proliferative and anti-neoplastic effects of selenium compounds.

Several selenium compounds are perfect candidates as chemotherapeutics since they are, to a certain extent, tumor selective, both in terms of their uptake and also in terms of their interactions with the unique pro-survival environment within or in the microenvironment of tumor cells. There is, thus, a true “selenium paradox in cancer” as features of the drug resistant cancer phenotype facilitate the cytotoxic action of the redox active selenium compounds.

This review aims at clarifying the importance of the chemical selenium species selected since the physiological and pharmacological effects are completely dependent on the relative redox reactivity often ignored by investigators. We further aim at explaining why several selenium compounds do have a marked pharmacological potential. Although these remarkable effects have been known for more than a century, there are very few clinical trials and until now, to our knowledge, no systematic phase I trial that would make systematic selenium therapeutic evaluation trials possible.

## 2. Selenium—An Antioxidant with Strong Pro-Oxidant Properties

Oxidation-reduction reactions are critical for the maintenance of the physiological homeostasis both in unicellular and multicellular organisms. The term “oxidative stress” is defined as the perturbations of the physiological redox homeostasis when the rate of cellular reduction is overwhelmed by the rate of cellular oxidation [5]. Both the enzymatic and non-enzymatic antioxidants can reverse this balance when the cellular damage is not beyond repair. In the context of selenium, the antioxidant properties are predominantly exerted by its incorporation into selenoproteins that can catalyze the reduction of disulfide bonds in proteins and peptides [6,7]. Such reductive properties are important for protecting against indiscriminate oxidation of intracellular and extracellular constituents by intrinsic and extrinsic oxidants, spanning the spectrum of disease conditions to harmful chemicals. Therefore, deciphering the physiological roles of selenium as an antioxidant is one of the key research areas that have been investigated and reinvestigated ever since the discovery of selenium as an essential trace element by Schwarz and Foltz in 1957 [8].

There is a broad diversity in nature of chemical species of selenium due to its four different oxidation states (−2, 0, +4 and +6). Interestingly, all selenium compounds with their different oxidation states are implicated in increased selenoprotein expression in *in vitro* and *in vivo* investigations; hence all oxidation states are potentially bioavailable for selenoprotein biosynthesis. These chemically diverse selenium compounds are utilized for physiological functions by unique metabolic pathways for each compound with variable degrees of overlapping intermediate metabolites. While hydrogen selenide is one of the major intermediary metabolites of inorganic selenium compounds, methylated selenium compounds (described in details in later section) are the major intermediary metabolites of organic selenium compounds [9,10]. On the basis of an increased capacity of selenoprotein synthesis by both of these classes of selenium compounds in eukaryotes, it is plausible that they are metabolized into a common pool of hydrogen selenide. Hydrogen selenide is subsequently converted into seleno-phosphate by seleno-phosphate synthetase 2 [11], a key step for the biosynthesis of selenocysteine and selenoproteins [12].

There is a stringent physiological regulation on the extensive capacity of selenoproteins biosynthesis in eukaryotes upon selenium supplementation in any chemical form. An enzymatic and protein saturation effect is found at optimum supplementation levels. Under this condition, any excess supply of selenium results in increased metabolism, but marginal or no further increases in selenoprotein biosynthesis. Many of these metabolites including hydrogen selenide and monomethylselenol are highly redox reactive and generate reactive oxygen species (ROS) upon reaction with and oxidation of thiols [13]. These compounds are therefore termed as redox-active selenium compounds (e.g., selenite, selenocystine, methylseleninic acid, Se-methylselenocysteine, *etc.*) that are known to exert oxidative stress [13]. Such pro-oxidative properties reflect the opposite spectrum of a common consensus that selenium is just an antioxidant [14]. It may, therefore, be clarified by selenium researchers, when so ever applicable, that redox-active selenium compounds are not antioxidants by themselves, but only when incorporated into selenoproteins with oxidoreductase functions and supplied at dietary dose levels corresponding to physiological optima.

**Figure 1:**
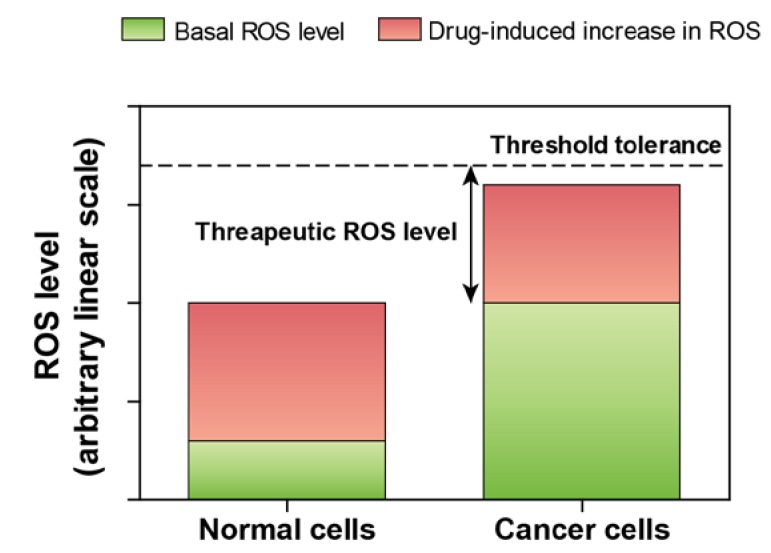
Effect of elevated level of ROS in normal and cancer cells.

From the above discussion, it is obvious that supplementation of supra-physiological levels of redox-active selenium compounds is necessary for induction of oxidative stress. An interesting question pertaining to such unfavorable biological effect is what implications this might have within the context of human health and disease? Although no obvious benefits may be conceived of by the damaging nature of ROS in a healthy condition, a potential application in the treatment of cancer can be harnessed, as there is a significant and emerging interest in the recent development of cancer therapeutics based on ROS-mediated mechanisms of action [15,16,17]. The underlying concept relies on the observations that cancer cells usually exhibit high basal levels of ROS compared to normal cells. Increased basal ROS levels and concomitant up regulation of antioxidant defense systems are pathophysiological adaptations of cancer cells favoring malignancy [16]. However, cancer cells have a lower tolerance to increased levels of ROS compared to normal cells [15,18], thereby providing a plausible window for therapeutic interventions by redox-active selenium compounds (Figure 1) [19,20].

## 3. Acute and Chronic Selenium Toxicity

To understand the anti-neoplastic mechanisms of redox active selenium compounds, its toxicity needs to be clarified. There are several reports of chronic and acute toxicity in man and animals, of natural, accidental and suicidal causes. A recent incident with fatal consequences happened in April 2009 where probably the largest test of acute selenium toxicity was unknowingly carried out in Venezuelan Polo Horses. The prized Lechuza Caracas horses were given an IV injection of Biodyl, a complex of vitamins and minerals used in Europe to increase stamina and reduce recovery time from fatigue in horses. The Biodyl formulation, unapproved in the United States by the Food and Drug Administration, was formulated in a pharmacy in Ocala, Florida. Several hours before the polo event, the horses were given an IV injection of Biodyl. Within a short period of time the horses began to collapse and within a few hours 21 of the prized Polo horses were dead.

The cause of death of the horses was reported to be owing to a misformulation of the Biodyl administered to the horses prior to the Polo match. The ensuing investigation focused upon the compounding pharmacy and the discovery that a mistake had been made in the amount of the sodium selenite component of the Biodyl formulation. The formulary chemist had mistakenly added ten times the amount of selenium that should have been contained in the Biodyl later injected into the horses. The Biodyl formula normally called for 500 µg of sodium selenite per mL of formulary but the amount of selenium from the pharmacy contained 5000 µg of sodium selenite per mL in the Biodyl formulation. News agencies reported that the fatal oral dose of sodium selenite to horses is 3300 µg or 3.3 mg/kg per body weight.

The amount of Biodyl injected into these horses by volume remains uncertain from the news reports. However, amounts of sodium selenite can be estimated from the treatment guidance for horses given from the Biodyl website. For horses, the French recommendation is for from 4–5 Biodyl IV administrations of 20 mL of Biodyl; each 4–5 h prior to the tournament or event. Thus these horses, by assumption, may potentially have received IV up to 20–25 g of selenium as sodium selenite within a relatively short period of time. While this is almost equivalent to the dietary toxicity of sodium selenite reported for horses, the fact that it was likely administered IV would make the sodium selenite much more toxic. The likely cause of death would have been massive erythrocyte hemolysis and suffocation consistent with the reported symptoms of the events at the time.

**Figure 2:**
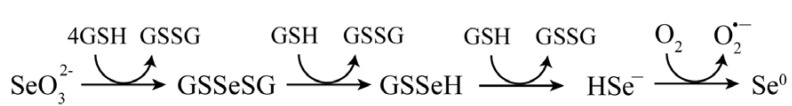
Reaction of selenite with glutathione and subsequent generation of superoxide anion, adapted from [2].

**Figure 3:**
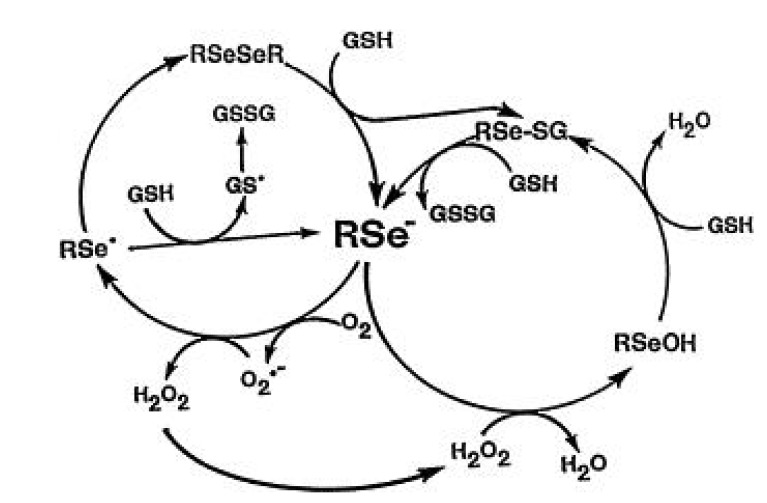
Redox cycling of selenium intermediates in the presence of GSH. Adapted from [27] and later republished [30]. Presented with permission from Taylor and Francis Group.

While the Polo horse deaths were indeed acute and tragic, chronic selenium toxicity in horses was also likely described by Marco Polo, *circa* 1265, while traveling the “Silk Road” in China [21]. Chronic selenium toxicity among horses and other range animals was reported and identified as such in the western United States in the 1930s. The differences systemically between acute and chronic selenium toxicity is dependent upon the dose, time and chemical species of the selenium compound either injected, as in the Polo horses, or as ingested by horses in the open range. It does seem that a common underlying effect of selenium toxicity, based upon a great deal of *in vivo* research with cells and in animals, is selenium’s ability as discussed below to catalytically oxidize thiols generating oxidative stress [22,23]. Selenium compounds of sufficient concentration to generate oxidative stress from thiols (RSH) must it seems have to be always present as a selenide or selenolate anion (RSe¯), as in sulfides (RS¯). Such anions of selenium are formed from the reaction of selenite with glutathione or other thiols. They initially generate a selenotrisulfide (RSSeSR), as initially proposed by Ganther [24] and upon further reduction form an unstable reduced selenopersulfide; RSSeH, ionizing at physiological pH forming the selenopersulfide anion, RSSe¯ [25]. This selenide catalyst oxidizes non-stociometrically additional thiols present cycling electrons through the selenium anion from thiols to oxygen forming superoxide, as originally proposed and shown below by Seko [26], for selenite (Figure 2) and later by Chaudière *et al.* for organic diselenides (Figure 3) [27]. It is from such reactions that the oxidative stress causing toxicity and the death of the Polo horses from selenite likely involved extensive hemolysis. The selenopersulfide anion catalyst is seen in reactions with selenite and GSH; and as the selenide or selenoate anion upon reduction in many diselenides experimentally using luminometry that measures superoxide [28]. Toxicity of selenium compounds was seen in early oral comparisons of selenium toxicity in mice, from a large library of mono- and diselenides as reported by Schwarz [29]. Almost without exception, organic diselenides were more toxic to mice than the corresponding monoselenides, due to the relative ease of diselenides being reduced to two catalytic organic selenides not readily occurring with monoselenides, (RSeR).

## 4. Redox Active Selenium Compounds and Detection of Superoxide

As noted above, selenium compounds are toxic based upon their chemical form, the dose and the biological exposure time. At nutritional levels of selenium intake and with a TUL of 400 µg Se/day, dietary selenium or supplemental selenium is not toxic to humans. In 1989, Seko *et al.* [31] were the first to suggest that the catalytic activity of selenite having been observed earlier by Feigal and West [32], by Tsen and Tappel, [33] by Rhead and Schrauzer [34] and later confirmed by Spallholz and Whittam [35], was likely toxic because it oxidized thiols and generated superoxide from GSH as shown in Figure 2 above by Seko and Imura [26].

The chemistry above is easily quantitated by the measurement of superoxide, the actively generated end product of the reaction. The superoxide so generated can be quantitated by a number of assays including cytochrome c [36] and methylene blue reduction [34] using visual spectroscopy. The superoxide electron may also be “trapped” and measured by ESR, Electron Spin Resonance Spectroscopy. We use a chemiluminescent cocktail of GSH and Lucigenin (bis-*N*-methylacridinium nitrate) and a water-jacketed luminometer to routinely measure superoxide. Additionally, quantitatively superoxide dismutase (SOD) [28,37] as well as iodoacetate, quenches the above and similar selenide or selenoate reactions with thiols confirming the generation of superoxide. *In vitro* and/or *ex vivo* a probe for superoxide, dihydroethidium (DHE) and its florescence, comes from the reaction of DHE with superoxide; Lambda Ex, 380 nm; 500 nm, Lambda Em, 580 nm. Cells treated with DHE from the reaction with superoxide will appear red in color when intercalating with DNA. The reaction can be semi-quantitated visually and fully quantitated using a fluorescence plate reader.

In 1992, Chaudière *et al.* [27] showed that for the diselenide selenocystamine, reduction of selenocystamine by GSH produced two molecules of redox active selenides that redox cycled in the presence of GSH. The organic diselenides, as shown in Figure 3 from Spallholz *et al.* [30] below, like selenite, generates superoxide while consuming oxygen, and these authors likewise concluded that this redox chemistry of organic diselenides, as proposed for selenite by Seko *et al.* [31], likely accounts for selenium’s toxicity.

**Figure 4:**
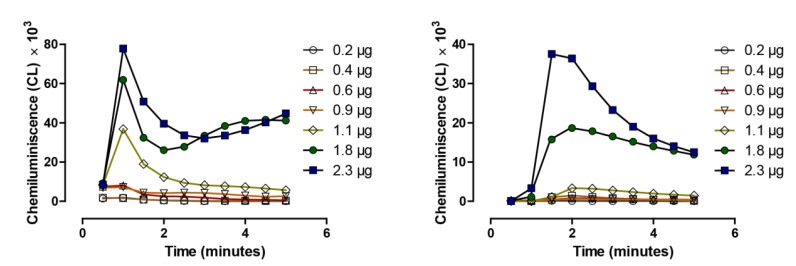
Quantitative analysis of generation of ROS, superoxide, by selenodiglutathione (**left**) and by selenite (**right**) in a lucigenin chemiluminescence-based assay, adapted from [38].

The reaction of selenocystamine as shown in the above equation in Figure 3 has also been extended to other aliphatic and aromatic diselenides. The catalytic selenium species in Figure 2 is likely to be GSSe-, a selenopersulfide, as selenodiglutathione (on left), the intermediate of his equation is more catalytic in a comparative chemiluminescent assay with selenite (on right) when equivalent amounts of selenium are added to equal amounts of GSH, (Figure 4) [38].

Superoxide is being measured in both panels of Figure 4 under conditions as in the Seko equation (Figure 2). On the left is the reaction with selenodiglutathione, and on the right, with selenite, the chemiluminescence is being shown over 5 min (mean of *n* = 3). The selenium, GSH concentration and all other parameters are the same in both assays. Note the chemiluminescent maxima are reached with GSSeSG nearly a full minute prior to being reached with addition of selenite. Total chemiluminescence generated by GSSeSG is also greater at all thiol concentrations in comparison to selenite. Our explanation for these differences is the time lag required for selenite to react with GSH forming GSSeSG, and the GSSe¯ catalytic anion, already preformed when GSSeSG is added to the reaction, requiring only a single reduction to the GSSe¯ anion, which we purpose as the catalytic species of the Seko equation. Both reactions with either GSSeSG or selenite eventually terminate with the formation of elemental selenium as shown by Seko (Figure 2) [26].

**Figure 5:**
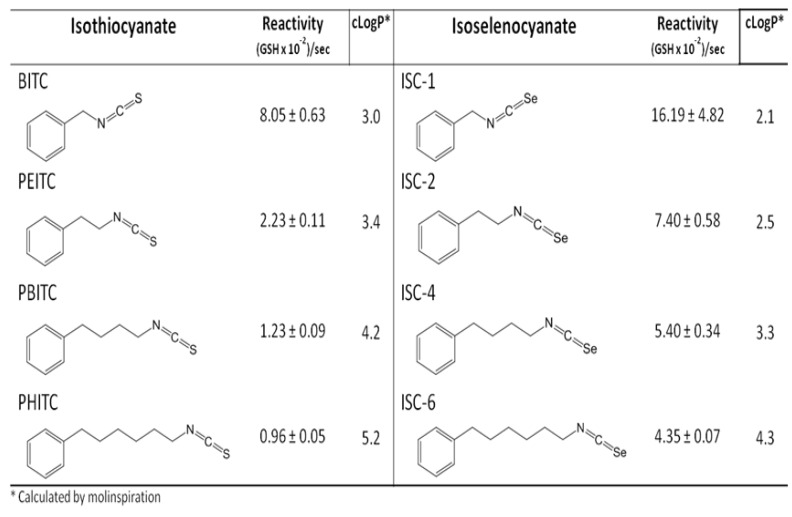
Comparative catalytic oxidation of GSH by isothiocyanates and isoselenocyanates, adapted from [39].

In addition to almost all selenium diselenides, selenium isoselenocyanates, (RNCSe) as well as isothiocyanates (RNCS) may continuously redox cycle in the presence of thiols and generate superoxide, as shown by Crampsie *et al.* [39] (Figure 5) without generating elemental selenium.

The redox activity of the reduced diselenides depends upon the R group moiety as it affects the oxidizing ability of the Se anion. The same is likely true for the isoselenocyanates (RNCSe) as well as the isothiocyanates (RNCS). In essence, both classes of the latter compounds likely have resonant features depending upon R that makes their analogs redox cycle. In any case, the selenium compounds, as shown with their sulfur analogs above are usually more redox active in generating superoxide than the corresponding sulfur analogs. A long held chemoprevention compound, sulforaphane, found in various cruciferous and other plant foods, is known to induce oxidative stress and upregulate Phase 1 and 2 enzymes, as does supplemental selenium [40]. It would be interesting to test sulforaphane *in vitro* in the chemiluminescent assay to see if, like the other isothiocyanates, it redox cycles, generating superoxide, as it is reported to do in human bronchial epithelial cells [41]. Many other authors have described either the toxicity and/or redox cycling of other selenium compounds and only some of these reports have been referenced here. The compounds include a number of modified organic compounds with selenium and most recently the emergence of seleno-nano particles containing selenite [42]. The authors believe that it can be concluded that those selenium compounds that generally redox cycle *in vitro* in any chemical spectral, fluorescence and/or chemiluminescense assay are very likely to be the more toxic selenium compounds to cells in culture and to animals *in vivo*.

As originally reported by Misra in 1974, the autoxidation of GSH spontaneously generates superoxide [43]. Therefore, across experimental time, researchers have consciously or unconsciously shown that selenium toxicity is simply manifested by the accelerated oxidation of GSH and other thiols generating superoxide and other reactive oxygen species. The actual death of the Polo Horses, so noted above, likely came from a combination of selenium and Fenton chemistry within the erythrocytes. A best guess scenario is selenite rapidly entered the horse erythrocytes generating superoxide and hydrogen peroxide. The iron being disturbed from hemoglobin induced Fenton chemistry, forming the highly toxic hydroxyl radical (OH˙) [44]. The addition of selenite to rat erythrocytes *in vitro* is known to cause hemolysis and cell destruction, as can be seen in scanning electron micrographs of the erythrocytes [45]. This redox chemistry can also be seen on polymer matrixes, where selenides are covalently or hydrophobically attached to cellulose, poly(methyl) methacrylate, or silicon. Such polymer surfaces coated with redox selenium become catalytic, generate superoxide in the presence of GSH, and visibly “oxidize” bacteria in their prevention of a biofilm formation on the polymer surface [46].

In addition to GSH and other low molecular weight thiols, the thioredoxin and glutaredoxin systems efficiently redox cycle with selenide or selenolates in the presence of oxygen [1,2,3]. These redox systems also reduce selenite and GSSeSG to selenide and to a limited extent reduce selenium from its highest oxidation state, selenate. Thus, selenium compounds that have a free selenolate or selenide, where a free selenolate might be formed, react widely within the cell with all accessible thiols and several redox systems, resulting in a non-stoichiometric consumption of thiols, NADPH and oxygen forming ROS and oxidative stress.

## 5. Methylation Reactions and Methylated Selenium Species

**Figure 6:**
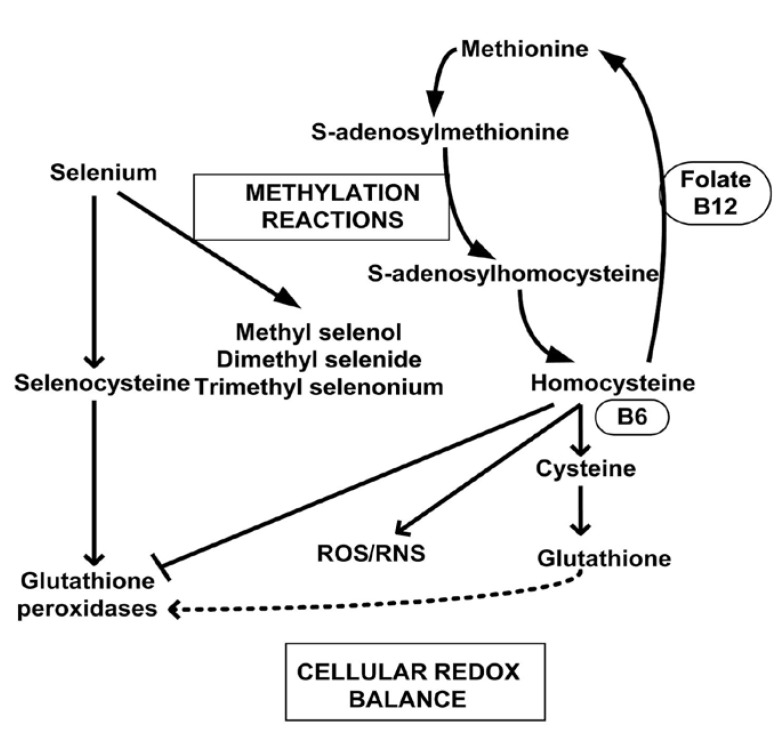
Important selenium methylation reactions and redox species in the maintenance of cell’s redox balance, adapted from [50].

In order to prevent selenium toxicity by selenoate or selenide formation in animals and cells, methylated selenides are synthesized forming mono- and dimethylselenide and a trimethylselenonium ion [47]. Complete or dimethylation renders any monoselenide non-catalytic, non-toxic, and, like other nutritional mono- and diselenides, they are ultimately and mostly excreted through the lungs or urine, following metabolism. The trimethylselenonium ion and Se-methylselenol-*N*-acetyl-galactosamine are major urinary metabolites of selenium [48]. Methylation of selenium is why the major dietary monoselenides, selenomethionine and Se-methylselenocysteine, do not generate superoxide *in vitro* and are not very toxic *in vivo*. Methylselenol, really methylselenide (CH_3_Se¯), is toxic and redox cycles when formed from reduced dimethyldiselenide, or when these two dietary amino acids are metabolically acted upon by gamma- and beta-lyases generating methylselenol; methylselenide. There is some consensus within the literature that the monomethylselenide is likely the chemoprevention/anti-cancer selenium metabolite [49] formed from the metabolism of selenomethionine, Se-methylselenocysteine or H_2_Se, which may also redox cycle as HSe¯, prior to being methylated as methylselenide. The metabolic pathway of selenium methylation and methylation reactions are shown in Figure 6 [50].

Methylation of some metals, non-metals and metalloids, which induce oxidative stress and are toxic, seems to be a general means of biological detoxification [51]. Mercury maybe the exception as inorganic mercury is less toxic than mono- or dimethyl mercury. Epigenetic control of genes is perhaps also influenced by the methylated dietary selenoamino acids and metabolic selenium intermediates [52].

## 6. Potent Antitumor Effects of Redox-Active Selenium Compounds

The redox reactivity of different selenium compounds has been described in the previous sections above. It seems obvious that the pro-oxidant functions of many of these redox-active forms of selenium are the key to their anticancer effects. We have recently documented the anticancer effects of selenite in detail (to be published soon). Herein we present the toxicity of two other important redox-active selenium compounds, Se-methylselenocysteine and selenocystine, and the rationale for their use as potential cancer chemotherapeutics.

## 7. Se-Methylselenocysteine

Se-methylselenocysteine (SMC) is not a standalone redox-active compound by itself. It is metabolized by glutamine transaminase K into methylselenol, which is highly redox reactive (described in earlier section). SMC is highly bioavailable, as indicated by increased SEPP biosynthesis and GPX1 activity upon supplementation, but relatively less toxic to cancer cells compared to other selenium compounds [53]. The reported 72 h IC_50_ values are 177 ± 32.2 µM and 100 ± 20 µM for HepG2 [53] and A549 cells [54], respectively. However, SMC is not very toxic to normal human cells (our own unpublished data). The significance of such observations indicates that it is possible to achieve a therapeutic selenium window. However, the *in vivo* therapeutic efficacy of SMC as an anticancer treatment in humans remains as an important question to be addressed.

Emerging data from animal studies indicate encouraging anticancer properties of SMC. In an orthotropic model of human colon carcinoma in athymic nude mice, it was shown that SMC reduces blood volume and micro vessel density in transplanted tumors [55]. Such inhibitory effects on angiogenesis were believed to be mediated via down-regulation of VEGF, COX2, HIF-1α and iNOS, as reported in an earlier study [56]. SMC not only inhibited angiogenesis, but also increased vascular maturation and reduced interstitial fluid pressure (reviewed in [57]). These effects were shown to be specific for tumor but not for normal organs, like the liver [58]. Such observations are suggestive of potentially increased chemotherapeutic drug delivery to the tumor without further increase in side effects. In fact, a recent study clearly demonstrates the remarkable antitumor capacity of SMC in combination with four different cytostatic drugs along with reduced side effects [59]. Certain combinations of SMC and cytostatic drugs resulted in complete remissions in an advanced xenograft model of Ward colorectal carcinoma in rats and human head and neck tumors in nude mice. Most importantly, SMC completely protected against Oxaliplatin-induced severe myelosuppresion in Fisher rats. Henceforth, the unique potential of this selenium compound strongly encourages further investigations on its application in the treatment of human cancer.

## 8. Selenocystine

Selenocystine (CysSeSeCys), is one of the key redox-active selenium compounds with dual functionalities—acting both as an antioxidant and a pro-oxidant. The parent compound itself does not exhibit any redox activity unless reduced by low molecular thiols and disulfide reductases to selenocysteine (Sec), which is highly redox active. Thus selenocystine is not significantly different than the reduction of selenocystamine, as shown in Figure 3. Under reducing condition, glutathione peroxidase-like activity of selenocystine has long been known [60]. Selenocystine is efficiently reduced to Sec by mammalian thioredoxin reductase with an apparent K_m_ and *k*_cat_ values of 6.0 µM and 3200 min^−1^, respectively [61]. Due to the lower pKa value of Sec, compared to cysteine, it is an efficient nucleophile. An excellent review on this subject has been published elsewhere [62]. The nucleophilic nature of Sec makes it very unstable, hence a potent reducing agent in its free form. The involvement of Sec insertion machinery in selenoprotein biosynthesis is probably one of the classical examples of evolutionary adaptation to harness Sec via aminoacylation of tRNA^[Ser]Sec^ thereby protecting the native form of Sec from its own nucleophilic activity prior to incorporation into selenoproteins.

**Figure 7:**
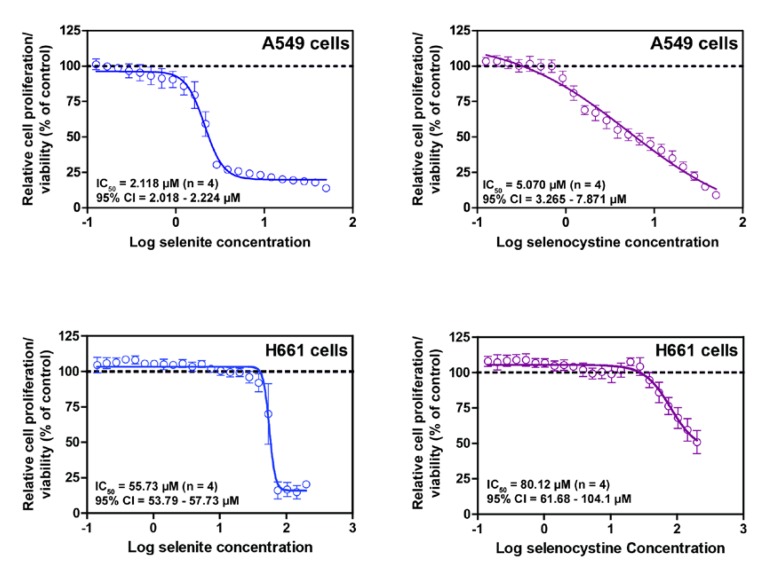
Cytotoxicity of selenite and selenocystine, as measured by a WST-1 assay kit (Roche, Mannheim, Germany), to A549 and H661 cells following 24 h exposure to 22 different selenium concentrations. The highest concentration of selenite and selenocystine was 50 and 200 µM for A549 and H661 cells, respectively. Note that due to low toxicity of selenocystine in H661 cells, the IC_50_ value should be interpreted carefully.

The redox potential of the intracellular milieu is conducive for efficient reduction of selenocystine. At nutritional levels, it may be presumed that selenocystine functions as an antioxidant with high bioavailability [53]. In spite of a potent electrophile inactivation capacity of Sec, selenocysteine is a potent toxic agent when present in excess. One of the key redox-active intermediate metabolites of Sec is hydrogen selenide that is generated by the action of the pyridoxal phosphate-dependent enzyme, selenocysteine lyase. Since this is a common metabolite of sodium selenite, one would expect a similar cytotoxicity profile of selenocystine and selenite when their uptake is similar. To address this, we performed cytotoxicity assays with selenite and selenocystine in A549 and H661 lung cancer cells. As shown in Figure 7, we found a remarkably similar cytotoxicity profile for these compounds. In another study, the 72 h IC_50_ data for these two compounds [63] corroborate very well with such assumptions. In this study, the authors noted higher sensitivity of selenite to normal cells compared to selenocystine (IC_50_ > 400 µM). Similar cytotoxic effects of selenocystine have been reported in mice thymic lymphoma cells, but not in isolated primary spleen lymphocytes [64]. This is somewhat suggestive of the cancer cell-specific effects of this compound. In a recent study, we have shown that selenocystine induces an unfolded protein response (UPR), ER-stress and large cytoplasmic vacuolization in HeLa cells [9]. The involvement of UPR as one of the key stress-associated pathway provides a possible mechanistic link between misfolded proteins and the disulfide reductase activity of Sec. It has been shown that selenocystine is less toxic compared to selenite at comparable doses in early postnatal male Wister rats [65]. Orally administered selenocystine is well tolerated in ICR mice when given up to 10 mg/kg body weight with no increase in plasma AST and ALT levels [66].

The pharmacokinetics data suggest that intravenous administration of this compound is very efficient in maintaining plasma selenium levels in comparison to oral administration in mice [67]. Early studies on DMBA-induced mammary carcinogenesis in BALB/c female mice showed limited protective effects of selenocystine on mammary adenocarcinoma [68]. The same study reported a significant lesser effect than selenite treatment. Comparative analysis indicates that this compound induces less DNA damage compared to selenite [69].

The most remarkable effects of selenocystine in humans were shown in the treatment of acute and chronic myeloid leukemia [70]. The authors reported greater effects of this compound on immature leucocytes than in mature leucocytes without any notable effect on bone marrow. Another interesting finding from the same study indicated increased response to selenocystine treatment in patients’ refractory to other chemotherapeutic agents then available. This recapitulates the findings from our own clinical trial study in which many patients who did not respond to cytostatic drugs, responded to subsequent selenite treatment (unpublished data, [71]). All the above studies clearly indicate that selenocystine may be a potential cancer chemotherapeutic candidate, especially for leukemias.

## 9. Selenium in the Treatment of Cancer—A New Era in Oncology

The applied dose and chemical species of selenium are the important aspects in the context of the development of tumor and neoplastic growth. Undoubtedly, selenium at low doses incorporated into enzymes and proteins function as an antioxidant for both normal and cancer cells. For the latter, optimum selenium anti-oxidant activity renders survival advantages to these genetically damaged cells during the progression and promotion stages of tumorigenesis. Nevertheless, selenium as an antioxidant protects against DNA damage that accumulates during the initiation phase of tumor development in a similar dose regimen. The augment of an antioxidant-based protective effect and improved immune functions by selenium are critical aspects for minimizing DNA damage and elimination of damaged cells by immune surveillance. Hence, the cancer chemopreventive effects of selenium as a single agent can only be predicted at physiologically relevant doses to evade DNA damage associated with carcinogenesis. In summary, it is the context that dictates the biological effects of nutritional levels of selenium at different stages of tumor development.

A purely cancer chemotherapeutic application of selenium requires higher doses of redox-active selenium compounds. Numerous studies have reported growth inhibitory and anti-neoplastic effects of selenium both *in vitro* and in animal models. Prior to the understanding of the pro-oxidative activity of selenite and diselenides, Greeder and Milner were much more successful in treating Ehrlich Ascites Tumor cells in mice with the later selenium compounds than with selenomethionine on a dose related basis [72]. Nevertheless, there are very few reports on the application of high doses of selenium for cancer treatment in humans. In the first half of the 20th century, colloidal selenium was successfully used for cancer treatment [73]. The evaluation of treatment efficacy is difficult to interpret from these studies, since the standard RECIST criteria were not available and the radiological methods were very primitive. A case series in 1956 reported favorable effects of selenocystine in the treatment of leukemia [70]. In two recent studies, moderate doses of selenite 0.2 mg/kg/day for 7 and 30 days, respectively, were administered together with standard cytostatic therapy with the primary aim to decrease side effects in patients with non-Hodgkin Lymphoma. The result was encouraging since the administration of selenite lead to a better response to therapy, apoptosis in lymphoma cells and a down-regulation of Bcl-2 [74,75]. Despite these promising reports, there has instead of a selenium treatment focus, there has been predominately only a focus of the possible preventive effects of selenium within the selenium research field. However, due to the failure of the SELECT trial [76] to prevent prostate cancer based on the use of selenomethionine and vitamin E, there has become confusion about the role and effects of selenium in the prevention of cancer. Several studies report beneficial effects by decreased side effects of the combination of low doses of selenium with cytostatic drugs and radiotherapy [77,78]. However, this possible clinical use of selenium and the preventive effects of cryotherapy are beyond the scope of the present review and will not be treated herein.

The major challenge in the use of selenium as a cancer therapeutic is to deliver redox active selenium, preferentially and directly to the tumor and/or metastatic cells. The most studied and simplest selenium compound, selenite must be administrated intravenously since no or very limited amounts of it will be detected in plasma after oral administration. We have recently completed a unique phase I trial in which the maximum tolerated dose, pharmacokinetics and safety of selenite was established (Unpublished data, [71]). The findings from this study will facilitate a long anticipated effect of selenite evaluation studies possible in humans. Selenite requires reduction prior to uptake and a reducing extracellular environment would facilitate its uptake [79]. Selenite might also be reduced by plasma thiols and there is possibly a complex kinetic mechanism where part of the administrated selenite will be reduced to elemental selenium [80]. Elemental selenium could serve as a storage form for further reduction to selenide. A similar metabolic transformation of selenite into elemental selenium has been shown in cancer cells [81] and in other animal species [82]. Selenomethionine is not suitable as a cytostatic drug since it is not redox active (rather a scavenger of ROS by auto-oxidation) and has a long biological half-life, due to its random incorporation in proteins in place of methionine, particularly albumin. Furthermore, although selenomethionine is a potential source of methylselenol (methylselenide) it is not applicable in humans due to a very poor activity of gamma-lyase.

Se-Methylselenocysteine (SMC) (see section of methylated selenium compounds) is a promising per oral candidate drug due to a very good bioavailability and the presence of active beta-lyases in human cells [83] (our unpublished data). This selenium compound is also one of the active organic constituents in selenized yeast. The anti-neoplastic action of SMC depends on the expression of beta-lyases. Only limited information is present regarding the expression of these highly interesting classes of enzymes in tumors. Further studies are ongoing to determine which tumor types SMC would be an efficient chemotherapeutic due to possible elevated expression of beta-lyases.

## 10. Novel Selenium Compounds and Future Perspectives for Therapeutic Selenium Drugs

The use of selenium in attempts to treat disease, particularly cancer, has a relatively long history dating back to at least 1912 when selenite was used to reportedly cure a tongue cancer. The literature contains many reports of selenium compounds, initially selenite in particular, but constantly being expanded to newer organic selenium compounds, arresting cancer cell growth *in vitro*. As noted above, some reports even suggest that selenium is more toxic to cancer cell lines than the more normal parent cells. It seems, on this basis, that the first reported successful prevention of prostate cancer in men was made by Clark *et al.* [84] using supplements of selenium-fortified yeast. When the major component of the selenium yeast, selenomethionine, was used in a larger human prostate cancer trial, the reported Clark outcome of the earlier trial was not, as noted, substantiated in the later larger human trial, SELECT [76]. Such prospective human trials, although few in number, and past animal cancer trials suggest to us that pure oral selenium supplementation of a normal selenium dietary intake will not prove to be broadly practical nor significantly beneficial to people with cancer ingesting adequate dietary selenium.

The authors do believe that it should be possible to target selenium’s toxicity for a more selective approach to treat diseases than by way of purely systemic supplementation. This approach today seems fully rational, as the toxicity of selenium compounds can be tailor-made and demonstrated to generate superoxide in cells *in vitro*. As in Figure 5 with the isothiocyanates and isoselenocyanates, it is empirically possible to design selenium compounds with organic functional groups that highly redox cycle and that can be covalently attached to improve anticancer drugs as in the case of Temozolomide [85], or attach redox selenium to monoclonal antibodies similar to T-DM-1, Kadcyla^®^ (ado-Trastuzumab Emtansine), in which Herceptin^®^ (Trastuzumab) carries the cytotoxic cytoskeletal drug, Emtansine. Over the course of the last decade, the authors and colleagues with whom we have collaborated have attached redox selenium to two vitamers, folic acid and biotin, both shown to redox cycle and are time and concentration dependently toxic to cancer cells *in vitro*. Together with graduate students, we have attached redox selenium to transferrin proteins [86] and monoclonal antibodies; Transferrin, Herceptin^®^ (Trastuzumab), and Avastin^®^ (Bevacizumab), all shown to be dose and time dependently more cytotoxic against cancer cells [87]. Such targeting of redox selenium offers the future prospect of using selenium toxicity in a more broadly beneficial therapeutic way.

## 11. Summary and Conclusions

Many selenium compounds are toxic, generating initially superoxide *in vitro* and thereafter other ROS *ex vivo* and *in vivo*. Toxicity is pH, selenium and thiol concentration dependent as measured for superoxide using chemiluminescense. Toxicity of selenite and organic diselenides, such as selenocystine, isoselenocyanates as well as aliphatic and aromatic selenocyanates are all positively or negatively affected by the R substitutions upon the selenide/selenonate anion. This information permits the synthesis of new selenium redox active compounds that may be added to existing drugs, or targeted with specific molecules, such as monoclonal antibodies. The modification of existing molecules with redox selenium, including nano-selenium containing particles is only limited by one’s imagination. One might even imagine a new era of selenium drug therapies [88].
